# Management of Preoperative Anxiety via Virtual Reality Technology: A Systematic Review

**DOI:** 10.3390/nursrep15080268

**Published:** 2025-07-25

**Authors:** Elina Christiana Alimonaki, Anastasia Bothou, Athina Diamanti, Anna Deltsidou, Styliani Paliatsiou, Grigorios Karampas, Giannoula Kyrkou

**Affiliations:** 1 Department of Midwifery, University of West Attica, 12243 Athens, Greece; e.alimonaki@yahoo.com (E.C.A.); abothou@uniwa.gr (A.B.); adiamanti@uniwa.gr (A.D.); adeltsidou@uniwa.gr (A.D.); 2 Second Department of Obstetrics and Gynaecology, Aretaieio University Hospital, National and Kapodistrian University of Athens, 11528 Athens, Greece; stpaliatsiou@yahoo.gr (S.P.); karampasgrig@gmail.com (G.K.)

**Keywords:** preoperative anxiety, Virtual Reality, adults, anxiety, surgery, nursing

## Abstract

**Background:** Perioperative care is an integral part of the procedure of a surgical operation, with strictly defined rules. The need to upgrade and improve some individual long-term processes aims at optimal patient care and the provision of high-level health services. Therefore, preoperative care is drawn up with new data resulting from the evolution of technology to upgrade the procedures that need improvement. According to the international literature, a factor considered to be of major importance is high preoperative anxiety and its effects on the patient’s postoperative course. High preoperative anxiety is postoperatively responsible for prolonged hospital stays, increased postoperative pain, decreased effect of anesthetic agents, increased amounts of analgesics, delayed healing of surgical wounds, and increased risk of infections. The use of Virtual Reality technology appears as a new method of managing preoperative anxiety. **Objective:** This study investigates the effect and effectiveness of Virtual Reality (VR) technology in managing preoperative anxiety in adult patients. **Methods:** A literature review was performed on 193 articles, published between 2017 and 2024, sourced from the scientific databases PubMed and Cochrane, as well as the trial registry ClinicalTrials, with a screening and exclusion process to meet the criterion of investigating VR technology’s effectiveness in managing preoperative anxiety in adult patients. This systematic review was conducted under the Preferred Reporting Items for Systematic Reviews and Meta-Analyses (PRISMA 2020) guidelines. **Results:** Out of the 193 articles, 29 were selected. All articles examined the efficacy of VR in adult patients (≥18) undergoing various types of surgery. The studies represent a total of 2.354 participants from 15 countries. There are two types of VR applications: distraction therapy and patient education. From the studies, 14 (48%) used the distraction VR intervention, 14 (48%) used the training VR intervention, and 1 (4%) used both VR interventions, using a range of validated anxiety scales such as the STAI, VAS-A, APAIS, and HADS. Among the 29 studies reviewed, 25 (86%) demonstrated statistically significant reductions in preoperative anxiety levels following the implementation of VR interventions. VR technology appears to manage preoperative anxiety effectively. It is a non-invasive and non-pharmacological intervention with minimal side effects. **Conclusions:** Based on the review, the management of preoperative anxiety with VR technology shows good levels of effectiveness. Further investigation of the efficacy by more studies and randomized controlled trials, with a larger patient population, is recommended to establish and universally apply VR technology in the preoperative care process as an effective method of managing preoperative anxiety.

## 1. Introduction

As we advance further into the 21st century, technological innovations continue to reshape the landscape of healthcare, including perioperative care. An estimated 312.9 million surgeries are performed worldwide each year, and the literature estimates the proportion of anxious patients at 25% to 80% in the Western world [1,2]. Perioperative management is a critical component of the surgical experience, encompassing a range of practices aimed at ensuring patient safety and optimal outcomes. One factor that is considered to be of significant importance is a high level of pre-operative anxiety and its impact on the patient’s postoperative course.

Anxiety is a distressing emotional state marked by worry, dissatisfaction, and fear. Physiologically, it activates the autonomic nervous system, including sympathetic, parasympathetic, and endocrine responses. These reactions can lead to elevated blood pressure and other stress-related symptoms. Biological stress markers such as cortisol in blood plasma and catecholamines in urine, along with vital signs like blood pressure and pulse, can be used to evaluate anxiety levels [3]. Preoperative anxiety is a common reaction of patients admitted for surgery caused by fear of the “unknown”, fear of an unsuccessful recovery after surgery, fear of unsuccessful surgery, anesthesia-related fear, fear of high postoperative pain, fear of not controlling the body’s functions during the operation, and fear of death [4].

Surgeries involving gynecological, oncological, and obstetric cases, performed for benign or malignant disease, cause additional stress and anxiety for women regarding the effects of cesarean section, loss of fertility, upcoming surgical menopause, insecurity due to loss of femininity and sexual function, self-image, functionality and survival combined with feelings of anxiety and loss of family in gynecological oncology surgery.

Female patients admitted for obstetric and gynecological surgery experience more preoperative anxiety than male preoperative patients, in comparison to the general population [5,6].

### 1.1. Effects of Stress on the Body Preoperatively and Postoperatively

Preoperative stress activates key physiological systems, including the hypothalamic–pituitary–adrenal axis and the sympathetic nervous system, leading to the release of stress hormones such as glucocorticoids and elevated pro-inflammatory cytokines [7]. These hormonal changes contribute to increased platelet aggregation, reduced heart rate variability, and the dysregulation of stress-response mechanisms [8]. Heightened adrenaline levels can interfere with anesthesia, decreasing its effectiveness and complicating dosage requirements. Moreover, stress weakens immune function and intensifies postoperative pain, which, in turn, may further suppress immune responses [9]. Common physical symptoms include elevated heart and respiratory rates, increased blood pressure, hyperglycemia, and gastrointestinal inhibition, all of which may hinder surgical preparation [10].

Unaddressed preoperative anxiety is associated with more severe postoperative pain, increased analgesic use, and higher risks of complications such as infections and delayed wound healing [3,9,11,12]. This can prolong hospitalization and hinder recovery, as stress contributes to hemodynamic instability and impairs tissue repair [13]. Psychoneuroimmunology research shows that stress alters the immune response and delays healing [9]. Additionally, stress-induced hyperglycemia, often seen in major surgery, is worsened by anxiety and negatively affects recovery and outcomes [14]. These findings underscore the importance of identifying and managing preoperative anxiety to improve the postoperative prognosis.

### 1.2. Need for Stress Management

Pharmaceutical treatments have historically been used to treat preoperative anxiety, but they have disadvantages, including the potential for adverse interactions with subsequent medications, temporary irritation of the mucosa, postoperative nausea and vomiting, and the development of delirium [15]. It is necessary to provide methods to reduce preoperative anxiety of non-pharmacological origin so that it does not affect the therapeutic outcome of surgery [3,13]. Guidelines should be developed and strategies should be implemented to reduce preoperative anxiety, particularly in obstetrics and gynecology. Women should be regularly reassessed during preoperative care and provided with appropriate means to reduce anxiety.

### 1.3. Technology of Virtual Reality

A range of non-pharmacological interventions have been explored as ways to help reduce preoperative anxiety, including music therapy, acupuncture, guided imagery, and relaxation techniques. While these approaches have been shown to be beneficial, they often involve passive engagement or require specific skills on the part of the practitioner. In contrast, Virtual Reality (VR) is emerging as a promising tool for managing preoperative anxiety by offering patients an immersive, interactive 3D environment [16]. Through a head-mounted display (HMD) with a 360° view and an embedded gyroscope for motion tracking, users can engage in real-time with calming virtual scenes, even while physically restricted during hospitalization [16,17,18]. This immersive distraction helps shift focus away from stress-inducing stimuli, temporarily reducing anxiety levels.

Ensuring patient safety remains essential in VR use. Guidelines developed by Monash University caution against its application in individuals with epilepsy, mental illness, pacemakers, or implanted devices due to potential risks from visual stimulation or radio waves [19]. As the technology becomes more portable, intuitive, and cost-effective, VR is increasingly adaptable for use in various clinical settings, enhancing its utility in both inpatient and outpatient care.

It is crucial to successfully manage pre-operative anxiety, promote mental well-being, and enhance the patient’s peri-operative treatment experience by concentrating on the patient and the best possible outcome of surgery. Two applications of VR are carried out to reduce anxiety: distraction therapy and patient education.

### 1.4. Distraction Therapy and Educational Use of VR

VR distraction therapy immerses patients in calming virtual nature environments—such as beaches, waterfalls, or forests—accompanied by relaxing music, helping alleviate anxiety through sensory engagement. In some cases, VR games have also been used for distraction [7].

The educational VR intervention provides patients with an immersive, informative tour covering all stages of the surgical journey—including relevant areas, procedures, and treatment methods to be followed. By virtually guiding patients through both the preoperative and postoperative process, this approach familiarizes them with what to expect and helps desensitize them to potentially stressful aspects of the experience. In some cases, the surgical procedure that will be used for the treatment of the patient’s condition will also be analyzed and virtually projected onto the patient. Preoperative uncertainty and anxiety can be reduced if participants have a detailed knowledge of what to expect before, during, and after the procedure [7].

Strategies that develop a knowledge-based patient education program, providing information, guidance, and full explanation regarding the interventional course, have been shown to effectively reduce preoperative and postoperative anxiety and increase positive attitude [20].

The field of VR is evolving rapidly. New technologies, improved hardware and software, and innovative applications of VR for health are constantly emerging. Previous systematic reviews have evaluated VR in healthcare settings. However, they have focused exclusively on randomized controlled trials (RCT)s, including studies published up to 2022, focused on pediatric populations, and data on adult surgical patients are limited.

A new systematic review would help update existing knowledge, incorporating the latest research data, and provide a more accurate and comprehensive picture of the effectiveness of VR. This review addresses an existing research gap by incorporating a broader spectrum of studies and more recent bibliographical data up to 2024. Consequently, it offers a more comprehensive understanding of the effectiveness of VR as a therapeutic intervention for reducing pre-operative anxiety.

### 1.5. Aim

This systematic review aims to collect, synthesize, and critically evaluate the available scientific evidence to gain an in-depth understanding of how VR can improve the patient experience before surgery by reducing preoperative anxiety and lead to better clinical outcomes.

Specifically, the objectives of this systematic review are as follows:

- To evaluate the effectiveness of VR interventions compared to standard care in reducing preoperative anxiety in adult surgical patients.
- To assess the impact of VR use on other perioperative outcomes, such as postoperative anxiety, patient satisfaction, medication use, and recovery time.
- To explore the feasibility and acceptability of VR among adult surgical patients, including ease of use, technical challenges, and patient satisfaction.
- To identify optimal parameters for VR intervention (e.g., content type, session duration, frequency) to guide future implementation and research.

By addressing these aims, the present review contributes valuable insights to clinicians, researchers, and healthcare policymakers regarding the integration of VR technology into perioperative practice for anxiety management.

## 2. Materials and Methods

### 2.1. PICO Eligibility Criteria

P—Population: Patients undergoing surgery.

I—Intervention: Use of VR as a preoperative tool. This includes VR-based educational videos and immersive distraction techniques administered before surgery.

C—Comparison: Standard care (e.g., routine verbal or written preoperative education, no intervention, or traditional distraction techniques), or in some studies, alternative non-VR educational tools.

O—Outcome: Reduction in preoperative anxiety levels, as measured by validated anxiety scales.

Based on this framework, the formulated research question was as follows: “In adult surgical patients, does the use of VR reduce preoperative anxiety compared to standard care or other non-VR interventions?”

### 2.2. Study Selection

The present systematic review was based on the PRISMA method (Preferred Reporting Items for Systematic Reviews and Meta-Analyses), and aggregated data published in international literature. A systematic bibliographic search was conducted across the electronic databases PubMed and Cochrane, the ClinicalTrials.gov registry for ongoing clinical trials, and the UpToDate clinical reference resource. Our search strategy follows recommendations suggesting that a minimum of two electronic databases is adequate for comprehensive literature coverage as per Atkinson & Cipriani, 2018 [21]. Study selection was guided by predefined inclusion and exclusion criteria, as outlined below.

### 2.3. Inclusion Criteria

(1) Patients who were scheduled to undergo surgery.
(2) Studies related to anxiety management.
(3) Studies using VR as a means of stress management.
(4) English-language articles.
(5) Full-page articles.

### 2.4. Exclusion Criteria

(1) Articles studying the VR intervention in children and adolescents.
(2) Articles that did not study VR exclusively (they also included other non-pharmacological interventions such as music therapy, aromatherapy, etc.).
(3) Articles that were diagnostic procedures (not surgeries) in an outpatient office location with or without local anesthesia.
(4) Protocols, feasibility studies, and pilot studies without full outcome data.
(5) Studies that did not report quantifiable anxiety outcomes (i.e., those lacking validated anxiety scales or measurable anxiety data).

### 2.5. Search Strategy

The search was based on the Eligibility Criteria of Participants, Intervention, Comparator, and Outcomes (PICO), using a combination of the following terms:

((((preoperative patients) OR (surgical patients)) AND ((virtual reality) OR (VR intervention))) AND (((preopera* inform*) OR (preopera* educ*)) OR (standard care))) AND ((preoperative anxiety) OR (preopera* anxiety)).

We performed a screening process from 2017 until 2024, to meet the criterion of investigating the VR technology effectiveness in the management of pre-operative anxiety in adult patients. The first search results were initially screened by two reviewers (ECA and AB), and after duplicates were removed, the remaining studies were independently screened according to the inclusion criteria also by two reviewers (ECA and AD) based on the title and abstract of potentially relevant articles. Then, articles were assessed based on the full text. Disagreements about the inclusion of articles were resolved by discussion with a fourth author (GK) to reach a final decision. Only full-text articles published in English were selected for further assessment.

### 2.6. Methodological Quality Assessment

Data from the included studies were extracted using a standardized form developed by the authors. Extracted variables included the author and year of publication, study design, sample size, type of surgery, characteristics of the VR intervention (e.g., educational or distraction-based), anxiety measurement tools used, and pre- and post-intervention anxiety outcomes.

The risk of bias in all articles was assessed using the MMAT (Mixed Methods Appraisal Tool). The mixed method appraisal tool (MMAT; Hong et al., 2018) is a critical appraisal instrument developed for the evaluation stage of systematic reviews [22]. It was used to assess the methodological quality of the reviewed studies. The MMAT was selected on account of its capacity to appraise an array of research designs, encompassing quantitative RCTs and quantitative non-randomized ones. The results of the quality assessment of the studies using the MMAT are presented in Table 1.

According to the MMAT (Mixed Methods Appraisal Tool), the quality assessment results in (Table 1) categorize two studies as have a moderate risk of bias, with a 60% score (Flores A. et al. 2023 [31]) and (Aardoom JJ. et al. 2022 [36]), 23 studies as having a moderate to low risk of bias, with an 80% score (Drozdova et al. 2024 [23], Bidgoli et al. 2023 [24], Amiri et al. 2023 [25], Pandrangi et al. 2023 [26], Rougereau G et al. 2023 [28], Grab M et al. 2023 [29], Martinez-Bernal D. et al. 2023 [32], Abbasnia F. et al. 2023 [33], Hermans et al. 2023 [34], Ugras GA. et al. 2023 [35], Touil N. et al. 2021 [37], Baytar AD. et al. 2021 [38], Turrado V. et al. 2021 [39], Turan AZ. et al. 2021 [40], Chan JJI. et al. 2020 [41], Hendricks et al. 2020 [42], Noben L. et al. 2019 [43], Ganry L. et al. 2017 [44], Bekelis K. et al. 2017 [45], Wang Y et al. 2024 [46], Asiri et al. 2024 [47], Schmid et al., 2024 [49], El Mathari et al. 2024 [50]), and four studies as having a low risk of bias, with a 100% score (Liu Y et al. 2023 [27], Kwon H et al. 2023 [30], Chiu et al. 2023 [15], Akar et al. 2024 [48]).

The research questions were identified, the purpose and objective of the present bibliographic review were defined, the literature was searched, and the results were extracted and presented.

A total of 29 articles were selected. Exclusion criteria for the rejected articles were as follows: 24 articles were duplicated, 33 articles were in a research status (Trials), 67 articles studied the effectiveness of VR in children and adolescents, and 35 articles did not meet the investigational criteria such as no full-page articles and studies with incomplete and irrelevant articles that did not meet the original aim of the study. Also, were excluded articles that were diagnostic procedures (not surgeries) carried out in an outpatient office setting, such as outpatient hysteroscopy and abnormal uterine bleeding. Due to significant heterogeneity across study designs, surgical procedures, VR modalities, and anxiety measurement tools, a meta-analysis was not feasible.

## 3. Results

### 3.1. Study Selection and Measurement Scales

**Figure 1:**
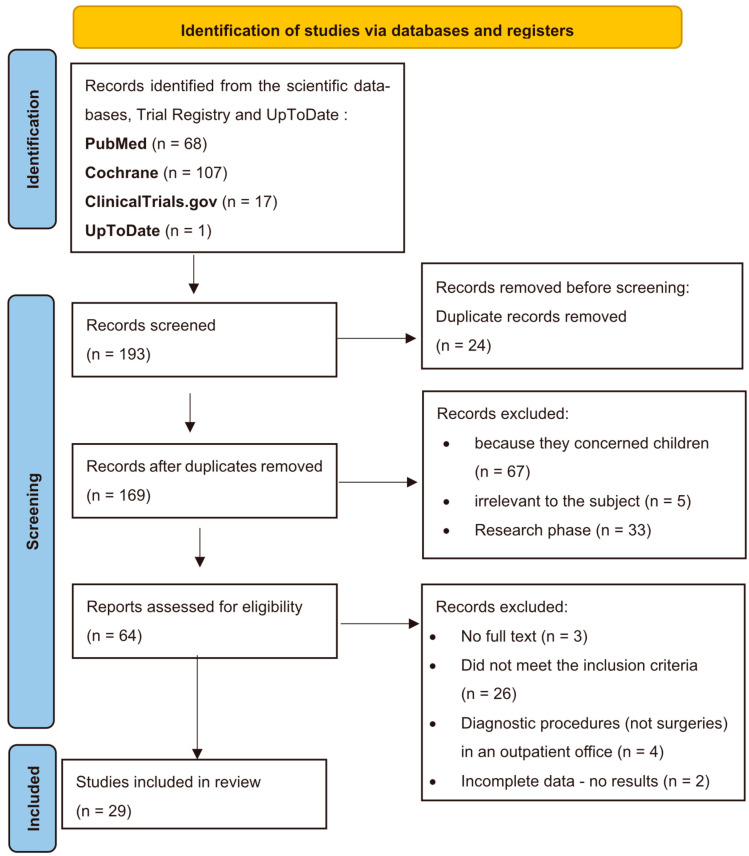
Flow diagram of study selection.

From the bibliographic search that has been performed, 193 articles were found. A total of 29 articles were selected. The selection of studies is illustrated in the flow diagram (Figure 1).

In most studies, there were two groups of patients to assess the effectiveness of VR. There was an intervention group with the standard preoperative preparation enhanced by VR and a control group that followed the standard preoperative preparation. Some of them used VR for all the participants. Preoperatively, at baseline and before VR intervention procedures, patients completed questionnaires based on some international anxiety measurement scales, and after the VR intervention, patients completed the same questionnaires again. The measurement scales used to measure anxiety specifically in the studies are listed in (Table 2 and Table 3). The most commonly used measurement scales for anxiety before and after the VR intervention are the VAS (Visual Analog Scale for anxiety), Spielberger State-Trait Anxiety Inventory (STAI), Amsterdam Preoperative Anxiety and Information Scale (APAIS), Hospital Anxiety and Depression Scale (HADS).

### 3.2. Characteristics of Included Studies

All articles examined the effectiveness of VR in adult patients (≥18) undergoing various types of surgery. The studies represent a total of 2.354 male and female participants from 15 countries: 11 studies in Europe, three in America, two in Australia, and 13 in Asia (Table 2). Among them, 18 studies included two groups, a VR group and a control group [Table 2 study number: 1-2-3-4-5-6-8-9-10-11-13-17-18-20-23-24-27-29], three studies included three groups (an education VR group, a distraction VR group, and a control group) [Table 2 study number: 7-12-16], seven studies applied the intervention in all patients [Table 2 study number: 15-19-21-22-25-26-28], and one study included a case report [Table 2 study number: 14]. Comprehensive details on the listed articles are given in (Table 2). Nineteen studies are randomized controlled trials—RCTs [Table 2 study number: 1, 2, 3, 4, 5, 6, 9, 10, 11, 12, 13, 16, 18, 19, 23, 24, 26, 27, 29].

### 3.3. Type of VR Intervention

**Figure 2:**
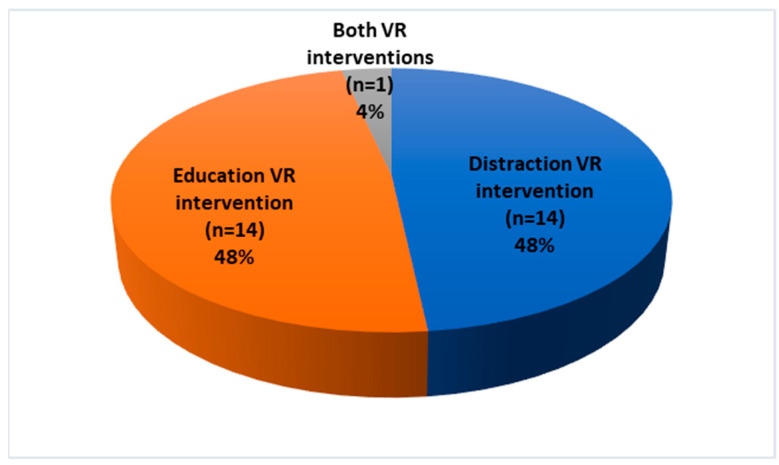
Intervention types of all included studies (n = 29).

The intervention types of all included studies (*n* = 29), are shown in the schematic diagram of intervention type in (Figure 2) and demonstrate that 48% used the distraction VR intervention, 48% used the training VR intervention and 4% used both VR interventions.

The most common assessment preoperative time was the day before surgery; other frequent ones were on surgery day. Some patients experienced VR during surgery under spinal anesthesia in two studies [Table 2 study number: 26,27]. The VR session length in 21 of the studies (Table 2) was between 5 and 30 min, with the most common time between 10 and 15 min. Also, a variety of immersive VR devices was used.

### 3.4. Adverse Effects

As far as the adverse effects are concerned, most of the studies had no adverse events. Out of a total of 2.354 participants, 11 patients reported adverse events and discontinued the VR procedure after experiencing one or a combination of the following symptoms: dizziness, nausea, claustrophobia, headache, blurred vision, eye pain, and eye fatigue. In the RCT by Liu Y et al., 2023 discomfort symptoms of five patients who faced mild adverse effects, including dizziness, nausea, and palpitations, were resolved with short breaks [27]. In all cases, the symptoms disappeared when the VR session ended and did not require any further treatment.

### 3.5. Quality Assessment

The risk of bias was assessed via the Mixed Methods Appraisal Tool (MMAT). All included studies were assessed and had acceptable selection criteria in their individual appraisal questions in the various domains, as per the quality assessment results (Table 1). However, some studies did not have a control group to compare the effectiveness of the intervention [Table 2 study number: 10-15-19-21-22-25-26-28] so the intervention was performed in the whole patient population. Even though without a control group, VR was shown to be effective because it reduced levels of preoperative anxiety from baseline, but there was no measure of comparison from a population of patients who followed the standard preoperative preparation procedure. In addition, some studies were conducted with a small number of participants. Blinding was not possible by participants due to the nature of the intervention, but blinding of outcome assessors and data analysts helped reduce detection bias. It should be noted that there was insufficient information on whether outcome assessors were blinded to treatment allocation in some of the included studies. This introduces a risk of bias in the outcome assessment. The randomization of patients in the intervention and control groups was verified by the researchers using suitable methods. In all studies, all patients who were assigned to the VR intervention completed the procedure, except for a few cases, clearly recorded in the studies, who discontinued the intervention for reasons such as urgent surgery, feelings of dizziness, and claustrophobia.

### 3.6. Effect of VR on Anxiety

The studies included in this systematic review displayed considerable variability in their sample characteristics, anxiety assessment tools, and reporting formats. Sample sizes varied widely across the 29 studies, ranging from a case report to larger RCTs. Anxiety was assessed using a range of validated scales, including the Visual Analog Scale for Anxiety (VAS-A), the Hospital Anxiety and Depression Scale (HADS), and the Visual Facial Anxiety Scale, among others. Pre-intervention anxiety levels were reported either as mean ± standard deviation (e.g., Wang et al., 2024: 8.38 ± 4.8) or as median with interquartile range (e.g., Akar et al., 2024: 3 (0–7)), depending on the underlying distribution of the data [46,48].

Post-intervention values generally indicated an anxiety reduction, as illustrated in studies such as Schmid et al. (2024), which reported a reduction from 3 (2–5) to 2 (2–3), and Wang et al. (2024), which showed a decrease from 8.38 ± 4.8 to 3.14 ± 3.9. A detailed summary of these outcomes is provided in Table 3 [46,49].

The timing, duration, and content of the VR intervention varied across the included studies, and there is a lack of consistent standards and guidelines. Most of the included studies examined the effect of VR technology on anxiety in patients suffering from a variety of health conditions, but there was no stratification by gender, age, type of anesthetic or type of surgery, which may have affected the results.

**Table 2:** Summary of relevant details of each included study.

	First Author, Country, Year	No of Patients	Scales Used	Intervention Protocol	Type of Surgery	Results
1	El Mathari et al., Netherlands, 2024 [50] [RCT]	121 patients. Control group (n = 33). VR group (n = 34).	(STAI) (APAIS)	(A) The VR group received educational information in the form of a virtual tour and simulation of the perioperative processes and areas, as well as a 3D animation providing a detailed explanation of the surgical procedure. (B) The control group received standard preoperative care.	Cardiac surgery	There was no difference in the levels of anxiety before surgery between the two groups. The level of satisfaction among patients regarding the information provided was found to be significantly higher among those in the intervention group.
2	Schmid et al., Australia 2024 [49] [RCT]	67 patients. Control group (n = 33). VR group (n = 34).	Visual Facial Anxiety Scale (VFAS)	(A) The VR group received standard preoperative care plus 3 min and 34 s of VR educational information through a virtual tour and simulation about the perioperative processes and areas. (B) The control group received standard preoperative care.	Gynecologic oncology Surgeries	Pre-operative anxiety was significantly decreased with VR. The VR intervention reduced anxiety immediately after its administration and also maintained the reduction in fear up until the time of surgery.
3	Akar et al., Turkey, 2024 [48] [RCT]	90 patients. Control group (n = 45). VR group (n = 45).	VAS-A SFQ	(A) The VR group watched 6.10 min of VR 360° video with nature sounds. (B) The control group received standard preoperative care.	Open-heart surgery	Nature sounds through VR effectively reduces preoperative surgical fear through SFQ but no statistically significant difference was found between the mean VAS-A scores of the intervention and control groups.
4	Asiri et al., Australia 2024 [47] [RCT]	95 patients. Control group (n = 45). VR group (n = 50).	APAIS VAS-A VRSQ VAS-P LPPSQ LOS	(A) The VR group received standard preoperative care plus 10 min of VR nature scenes, sounds, and music. (B) The control group received standard preoperative care. Also, cortisol levels and heart rate were measured.	Elective surgery	Pre-operative anxiety was significantly reduced with VR. However, VR also showed promise in improving postoperative outcomes.
5	Wang et al., Czech China 2024 [46] [RCT]	115 patients. Control group (n = 57). VR group (n = 58).	VAS HADS-A HADS-D STAI	(A) The VR group received standard pre-operative care plus 15 min of VR with scenes of nature, sounds, and music. (B) The control group received standard preoperative care.	Laparoscopic gynecology surgery	In female patients undergoing laparoscopic gynecological surgery, VR can reduce preoperative anxiety.
6	Drozdova et al., Czech Republic 2024 [23] [RCT]	150 patients—two groups of 75 people.	five-point Likert scale	(A) The VR group watched a 6 min 360° educational video about the procedure. (B) The control group received training from a doctor.	Permanent pacemaker implantation	VR education reduces patient anxiety 92%; it also improves patient understanding levels.
7	Bidgoli et al., Iran, 2023 [24]	105 patients—three groups of 35 people.	STAI	(A) In-person group visited the operating theatre for 30 min on the day before surgery. (B) The VR group watched a 30-min virtual tour of the operating room the day before surgery. (C) The control group received standard care.	Hernia, cholecystectomy, appendectomy, cesarean section, hysterectomy, hemorrhoidectomy	There was no significant effect on patients’ preoperative anxiety, nor was there any reduction in anxiety levels before and after the interventions.
8	Amiri et al., Iran, 2023 [25]	60 patients—two groups of 30 people.	STAI-	(A) The VR group watched a VR film. (B) The control group watched an ordinary video about the physical area and operating room, the day before the operation. Duration was 4 min and 35 s.	Open-heart surgery	The difference in anxiety levels between the VR and ordinary video groups after the intervention was significant.
9	Pandrangi et al., Oregon, USA, 2023 [26] [RCT]	32 patients—two groups of 16 people.	VAS	In Group 1, patients played a preoperative 15 min VR game and received a postoperative VR mindfulness experience, while Group 2 had the same interventions in the reverse order.	Head and neck surgery	Different VR experiences appear to be associated with similar reductions in perioperative anxiety in patients.
10	Liu Y et al., China, 2023 [27] [RCT]	114 patients. Control group (n = 57). VR group (n = 57).	SΤA-AI	(A) The VR group received a 16 min training video that informed patients about the procedures and was complemented by a sightseeing experience. (B) The control group used a tablet for viewing.	Carotid artery stenting (CAS)	Patients’ anxiety was reduced in both groups, but more significantly in the VR group.
11	Rougereau G et al., France, 2023 [28] [RCT]	60 patients—two groups of 30 people.	STAI	(A) The VR group had a 10 min VR distraction of landscapes (sea, beach, or forest). (B) The control group received routine care.	Hallux valgus surgery	A VR hypnosis mask before surgery modestly reduced postoperative and predischarge anxiety. There was a notable decrease in immediate higher-level postoperative analgesics such as morphine or ketamine.
12	Grab M et al., Germany, 2023 [29] [RCT]	99 patients. Control (n = 34). 3D-printed (n = 34). VR (n = 31).	VAS STA SAS TAS	Patient education methods were (A) information via printed leaflet (n = 34), (B) 3D printed models (n = 34), and (C) VR models (n = 31).	Coronary artery surgical aortic valve replacement, thoracic aortic aneurysm surgery, and bypass graft	The use of VR significantly reduced the anxiety and improved the understanding of the procedures. Visualization with 3D VR and 3D models contributed significantly to the result. Also, patient satisfaction with the new interventions was increased.
13	Kwon H et al., South Korea, 2023 [30] [RCT]	80 patients—two groups of 40 patients.	APAIS	(A) The VR group received preoperative education about preoperative and postoperative processes and their management. (B) The control group received preoperative education with traditional verbal education.	Reduction of facial bone fracture, corrective rhinoplasty, breast augmentation, skin or soft tissue excision, reconstruction, and burn	Compared to verbal education, patients’ anxiety successfully reduced by VR. The level of satisfaction in the VR group was also higher than that of the control group.
14	Flores A. et al., Switzerland, 2023 [31]	Case Report—1 woman.	GRSs STAI-Y	The patient spent 10 min with no VR vs. 10 min in VR distraction (the treatment order was randomized). There was a 3 min rest period after the first 10 min intervention.	Laparoscopic cholecystectomy	A 67% lower preoperative anxiety was observed during VR intervention.
15	Martinez-Bernal D. et al., U.S.A., 2023 [32]	30 patients (17 female and 13 male).	AIM FIM	Patients watched a VR distraction video for 2 min with natural scenes.	Oral—underwent a maxillofacial surgery	The preoperative use of VR was highly accepted by patients. Pre-operative anxiety appears to be statistically significantly reduced (*p* = 0.003).
16	Abbasnia F. et al., Iran, 2023 [33] [RCT]	150 patients—three groups of 50 people.	STAI	(A) Education group and (B) distraction group received two 5 min VR, 2 hours before and 4 hours after surgery. (C) The control group received standard care.	Laparoscopic cholecystectomy	A significant reduction in pre-operative anxiety observed in VR groups.
17	Hermans ANL et al., Netherlands, 2023 [34]	134 patients. Control (n = 66). VR (n = 68).	APAIS	(A) The VR group received educational information according to the procedures and standard preprocedural information. (B) The control group received standard preprocedural information.	Atrial fibrillation (AF) ablation	The VR educational video led to better provision of information and knowledge of patients. An increase in patient satisfaction was also observed and reduced anxiety.
18	Ugras GA. et al., Turkey, 2023 [35] [RCT]	86 patients—two groups of 43 people.	ASSQ SBP DBP HR RR SpO2	(A) The VR group received 10 min of five 3D videos with relaxing music. (B) The control group received standard care.	Colorectal and abdominal wall surgery	Anxiety levels were significantly reduced in the VR group and significantly increased in the control group after standard preoperative care. The VR intervention also reduced psychological and physiological responses to PA.
19	Chiu et al., China, 2023 [15] [RCT]	74 patients.	APAIS and VAS	(A) The VR group received an 8 min educational information video through a virtual tour and simulation about the perioperative process. (B) The control group received standard preprocedural information.	General surgery, functional endoscopic sinus surgery, and arthroscopic surgery	The use of VR can be effective in reducing pre-operative anxiety, stress, and preparedness in adult patients undergoing elective surgery.
20	Aardoom JJ. et al., Netherlands, 2022 [36]	Eight patients—two groups of four people.	PQ ITQ	All patients received VR for 20 min, some at the hospital and some at home. The VR content was educational information through a virtual tour and images related to cardiac catheterization.	Cardiac catheterization	VR was reported to be effective, contributing to stress management and improving patients’ knowledge of the care process. Negative psychological outcomes after the procedure were reduced.
21	Touil N. et al., Belgium, 2021 [37]	48 patients.	APAIS APAIS-A and APAIS-S VAS	All patients experienced a VR clinical hypnosis session for 15 min, with suggestions for muscle relaxation and deep breathing under relaxing music.	Elective hand surgery under local anesthesia	The total anxiety score was significantly reduced with VR.
22	Baytar AD. et al., Turkey, 2021 [38]	40 patients.	STAI	All patients received VR for 15 m, with distraction content of nature and meditation music.	Septorhinoplasty	There was a significant decrease in preoperative anxiety with VR application.
23	Turrado V. et al., Spain, 2021 [39] [RCT]	126 patients—58 exposed and 68 unexposed.	STAI-S HADS	(A) The VR group experienced a realistic environment educative and informative for 16:34 min, which was created in the facilities of the Hospital. (B) The control group received standard information	Colorectal cancer surgery	After exposure, all anxiety/depression rating scales showed a significant decrease.
24	Turan AZ. et al., Turkey, 2021 [40] [RCT]	97 patients;—50 patients in the study group and 47 in the control group.	STAI-TA STAI-SA VAS	(A) The VR group, after spinal anesthesia and during the operation, watched a film with VR glasses. (B) The control group, also under spinal anesthesia, followed the standard procedure without VR.	Lower abdominal, anogenital, urologic, and lower extremity surgeries	VR during surgery reduces perioperative anxiety under spinal anesthesia.
25	Chan JJI. et al., Singapore, 2020 [41]	108 patients.	HADS	All patients received a 10 min VR intervention consisted of natural scenes, background meditation music, and breathing exercises.	Minor gynecological surgeries	Statistically significant reduction in pre-operative anxiety and depressive symptoms with VR intervention reported.
26	Hendricks TM. et al., Mayo Clinic in Rochester, Minnesota, U.S.A., 2020 [42] [RCT]	20 patients.	STAI	(A) The VR group received both interventions for 20 min each. (B) The control group used a tablet-based game application with audiovisual stimulation.	Cardiac surgery	In the VR group, a superior and significant improvement in the feeling of calmness and a significant reduction in the feeling of stress were observed.
27	Noben L. et al., Netherlands, 2019 [43] [RCT]	97 women (49 exposed VR and 48 unexposed).	VAS-A SSQ CPS PCQ	(A) The VR group received educational oral information and VR. (B) The control group received standard information from the doctor.	Cesarean Delivery	No significant decrease in VAS-A score in the VR group (n = 49) in comparison to the control group (n = 48). After viewing the VR video, the VR group reported feeling more prepared for the procedure.
28	Ganry L. et al., France, 2017 [44]	20 patients (10 men and 10 women)	APAIS. salivatory cortisol heart coherence (HC)	All groups were virtually immersed in nature for 5 min. There was a wide range of themes available for patients to choose from, which are beneficial for relaxation.	Skin cancer surgery	There was a significant reduction in the VAS score after the VR (*p* < 0.009) as was the level of salivary cortisol (*p* < 0.04). Cardiac coherence values remained unchanged
29	Bekelis K. et al., Lebanon, 2017 [45] [RCT]	127 patients—64 in the VR group and 63 in the control group.	EVAN-G APAIS	(A) The VR group watched a 5 min VR educative video about the preoperative and postoperative experience on the day of the surgery. (B) The control group had routine audiovisual education. In addition, a physician verbally explained the preoperative experience to them.	Elective craniotomy or spine surgery	Patients who were exposed to the VR experience had a higher level of satisfaction during the preparation for surgery. Also, the VR group had less anxiety in the perioperative period.

Abbreviations: Virtual Reality (VR). Rating scales used in the studies: Visual Analog Scale for anxiety (VAS); Visual Analogue Scale for pain (VAS-P); Virtual Reality Symptom Questionnaire (VRSQ); Hospital Length of Stay (LOS); Leiden Perioperative Patient Satisfaction Questionnaire (LPPSQ); Spielberger State-Trait Anxiety Inventory (STAI); Amsterdam Preoperative Anxiety and Information Scale (APAIS); Hospital Anxiety and Depression Scale (HADS); Anxiety Specific to Surgery Questionnaire (ASSQ); State Anxiety Inventory (S-AI); State-Anxiety-Score (SAS); Trait-Anxiety-Score (TAS); Acceptability of Intervention Measure (AIM); Feasibility of Intervention Measure (FIM); Surgical Fear Questionnaire (SFQ); Graphic Rating Scales (GRSs); Evaluation du Vecu de l’Anesthesie Generale (EVAN-G); Simulation Sickness Questionnaire (SSQ); Childbirth Perception Scale (CPS); Pregnancy and Childbirth Questionnaire (PCQ); State-Trait Anxiety Inventory for Trait Anxiety (STAI-TA); State-Trait Anxiety Inventory for State Anxiety (STAI-SA); Presence Questionnaire (PQ); Immersive Tendencies Questionnaire (ITQ); systolic blood pressure (SBP); diastolic blood pressure (DBP), heart rate (HR); respiratory rate (RR); and peripheral oxygen saturation (SpO2).

**Table 3:** Results of Virtual Reality in the management of preoperative anxiety from the included studies.

	Study	Age (Years)	Pre-Intervention Anxiety (Mean ± SD/Median [IQR])	Post-Intervention Anxiety (Mean ± SD/Median [IQR])	Anxiety Rating Scales	*p*-Value
1	Schmid et al., 2024 [49]	57.0 ± 13.9	VR: 3 [2–5]; Control: 4 [2–5]	VR: 2 [2,3]; Control: 4 [3–5]	Visual Facial Anxiety Scale	Statistically significant reduction of anxiety *p* < 0.001
2	Akar et al., 2024 [48]	63.79 ± 8.26	VR: 3 [0–7]; Control: 2.2 [0–7.5]	VR: 2 [0–7.5]; Control: 2.9 [0.5–8.2]	VAS-A	No significant difference between the anxiety levels of the study groups (*p* > 0.05).
3	Wang et al., 2024 [46]	VR: 37.2 ± 7.2 Control: 38.7 ± 8.3	VR: 8.38 ± 4.8 Control: 9.74 ± 5.5	VR: 3.14 ± 3.9 Control: 9.81 ± 6.1	HADS	Statistically significant reduction of anxiety *p* < 0.001
4	Asiri et al., 2024 [47]	VR: 51 ± 15 Control: 44 ± 18	VR: 42 (±2.0), 38–46 Control: 59 (±2.1), 55–64	VR: 22 (±3.9), 14–29 Control: 29 (±3.9), 21–37	VAS-A	Statistically significant reduction of anxiety *p* < 0.001
5	Drozdova et al., 2024 [23]	76 (70–83)	Not available numerically.	Anxiety was reduced in 92% of patients (n = 69)	a five-point Likert scale	Not Available
6	Turrado et al., 2021 [39]	VR: 64 (41–85) Control: 68 (50–86)	HADS VR: 8.00 (7.00; 11.00) Control: 7.00 (6.00–8.00) STAI-S VR: 20.00 (17.00; 24.00) Control: 22.00 (19.00–24.00)	HADS VR: 5.00 (4.00–6.00) Control: Not measured STAI-S VR: 11.50 (7.00–14.00) Control: Not measured	STAI-S, HADS	Statistically significant reduction of anxiety *p* < 0.001
7	Martinez-Bernal et al., 2023 [32]	Not reported (categorical only)	VR: 5.82	VR: 3.96	FIM	Statistically significant reduction of anxiety *p* = 0.003
8	Flores et al., 2023 [31]	44 (case report)	“moderately anxious” (6 out of 10) “strong fear” (rated 8 out of 10)	Reported 67% lower presurgical anxiety during VR. “mildly anxious” during VR (2 of 10) “no fear” (0 of 10) during VR	STAI-Y	Not Available
9	Grab et al., 2023 [29]	64.86 ± 10.90	VAS: 5.00	VAS: 4.32 (Δ − 0.68)	VAS	Statistically significant reduction of anxiety *p* < 0.001
10	Abbasnia F. et al., 2023 [33]	43.85 + 11.78	VR1 distraction 43.04 ± 11.57 VR2 education 43.04 ± 11.57 Control 51.7.8 ± 15.88	VR1 distraction 34.97 ± 11.37 VR2 education 32.61 ± 9.88 Control 55.21 ± 20.01	SSAI	Statistically significant reduction of anxiety *p* < 0.001
11	Baytar et al., 2021 [38]	32.7 ± 8.7	STAI-S: 41.9 ± 5.7 40.5 (Median)	STAI-S: 35.1 ± 4.8 34 (Median)	STAI-S	Statistically significant reduction of anxiety *p* < 0.001
12	Ugras et al., 2022 [35]	VR: 44.7 ± 12.9 Control: 43.0 ± 15.8	VR: 30.9 ± 6.8 Control: 29.0 ± 5.8	VR: 25.1 ± 6.5 Control: 29.7 ± 6.2	ASSQ	Statistically significant reduction of anxiety *p* < 0.001
13	Chiu et al., 2023 [15]	46.34 ± 14.52	VR: 23.92 ± 2.19 Control: 23.03 ± 3.09	VR: 15.92 ± 4.67 Control: 20.59 ± 4.82	APAIS	Statistically significant reduction of anxiety *p* < 0.001
14	Amiri et al., 2023 [25]	56.1 ± 7.6	VR: 55.8; Control: 58.33	VR: 38.6; Control: 45.13	STAI	Statistically significant reduction of anxiety *p* < 0.001
15	Turan et al., 2021 [40]	43.8 ± 16.41	STAI-SA VR: 44 ± 10 Control: 42 ± 10 STAI-TA VR: 48.6 ± 6.7 Control: 47.49 ± 7.17	VR During operation: VAS VR: 4.1 ± 1.91 Control: 5.19 ± 1.64	STAI, VAS	Statistically significant reduction of anxiety *p* < 0.003
16	Bekelis et al., 2017 [45]	55.3 ± 14.0	APAIS-Difference (95% CI) -Stratified on type of operation 29.9 (24.5 to 35.2)	APAIS VR: 90.7 Control: 60.8 VAS 41.7 points lower in the VR group compared to the control group (–41.7 (–33.1 to –50.2))	APAIS, VAS	Statistically significant reduction of anxiety *p* < 0.01
17	Ganry et al., 2018 [44]	56.9	VAS 3.3 before the VR test	VAS 2.85 after the VR test	VAS	Statistically significant reduction of anxiety *p* < 0.009
18	Noben et al., 2019 [43]	32.6 ± 3.9	Control group 3.8 [SD 2.3] VR group 4.1 [SD 2.3]	Control group 4.6 (2.5) VR group 5.6 (2.4)	VAS-A	Νo statistically significant difference *p* = 0.08
19	Hendricks et al., 2020 [42]	VR: 69.5 ± 6.9; Control: 63.4 ± 9.1	STAI-TA VR: 36 ± 6 Control: 33 ± 8 STAI-SA VR: 19 ± 1.7 Control: 20 ± 2.0	STAI-SA VR: 15 ± 1.3 Control: 17 ± 2.0	STAI	Statistically significant reduction of anxiety *p* < 0.05
20	Liu et al., 2023 [27]	64.8 ± 11.3	STAI-SA VR: 47.9 ± 10.5 Control: 48.9 ± 9.5 STAI-TA VR: 46.7 ± 11.7 Control: 46.9 ± 9.9	STAI-SA Post-intervention VR: 40.1 ± 7.8 Control: 43.5 ± 8.5 STAI-SA Post-operation VR: 35.0 ± 6.2 Control: 38.3 ± 6.8	STAI-S, STAI-T	Statistically significant reduction of anxiety Post-intervention *p* = 0.036 Post-operation *p* = 0.014
21	Pandrangi et al., 2023 [26]	47.3 ± 16.7	Group 1 preoperative VR gaming VR: 3 (18.8) Group 2 preoperative VR mindfulness VR: 4 (25.0)	Group 1 postoperative VR mindfulness: −12.0 [15]; Group 2 postoperative VR gaming: −10.5 [13]	VAS-A	Different VR experiences appear to be associated with similar reductions in perioperative anxiety *p* = 0.62
22	Aardoom et al., 2022 [36]	67 ± 7.5	Not measured	4.0 (0.9) (7/8, 88%) reported VR to be effective. Questionnaire ranged from 1 (totally do not agree) to 5 (totally agree)	5 scaled questionnaire	Not Available
23	Touil et al., 2021 [37]	Median: 49 (range: 19–76)	APAIS-A VR: 7 (4, 8) VAS anxiety (0–10) VR: 5 (4, 7)	APAIS-A VR: 3 (3, 5) VAS anxiety (0–10) VR: 2 (1, 6)	APAIS, VAS	Statistically significant reduction of anxiety *p* < 0.001
24	Hermans et al., 2023 [34]	Median: 66 (range: 58–72)	VR: 11 (9–14) Control: 9 (6–12)	VR: 13 (19.1%) Control: 27 (40.9%) Less worries in VR (*p* = 0.006)	APAIS	Statistically significant reduction of anxiety *p* = 0.006
25	Kwon et al., 2023 [30]	40.75 ± 15.60	VR: 15.65 ± 1.96 Control: 15.85 ± 1.31	VR: 7.73 ± 1.52 Control: 13.00 ± 1.16	APAIS-A	Statistically significant reduction of anxiety *p* < 0.001
26	Chan JJI. et al., 2020 [41]	43.56 ± 6.68	7.2 ± 3.3	4.6 ± 3.0	HADS	Statistically significant reduction of anxiety *p* < 0.0001
27	Rougereau et al., 2023 [28]	55 ± 13	VR: 44.3 ± 5.8 Control: 46.3 ± 5.8	VR: 42.5 ± 9.7 Control: 45.2 ± 7.9	STAI	Statistically significant reduction of anxiety *p* < 0.04
28	El Mathari et al., 2024 [50]	67.88 ± 8.56	STAI (20–80) VR: 38.00 ± 11.99 Control: 39.21 ± 8.76 APAIS VR: 6.00 (4) Control: 6.00 (6)	STAI (20–80) VR: 39.90 ± 11.75 Control: 40.18 ± 9.51 APAIS VR: 5.50 (5) Control: 5.00 (4)	STAI APAIS	Νo statistically significant difference between the two groups (*p* > 0.05)
29	Bidgoli et al., 2023 [24]	40 ± 11	VR: 54.80 ± 4.63 Control: 55.62 ± 4.89	VR: 54.48 ± 5.04 Control: 53.42 ± 4.62	Spielberger’s anxiety questionnaire	Νo statistically significant difference between the two groups (*p* = 0.10)

Age values are reported as Mean ± SD or Median [IQR], depending on the original source. Studies that reported age only in categorical ranges are noted accordingly. For case reports, a single age value is provided.

**Figure 3:**
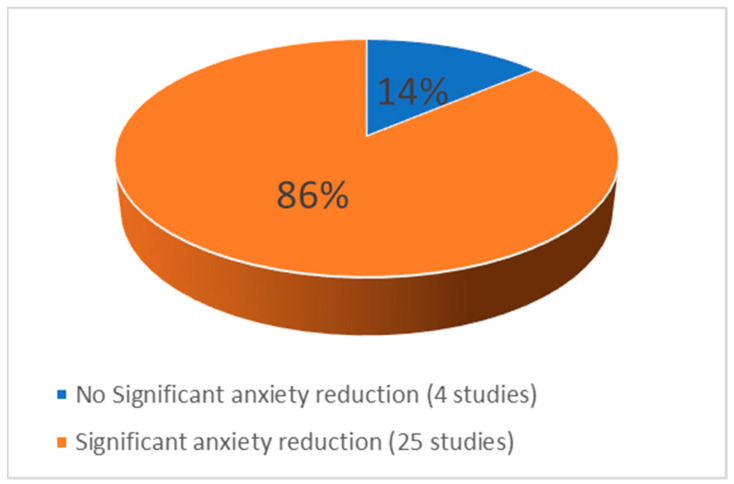
Effectiveness of VR from the included studies.

The overall findings, as summarized in Table 3, indicate that the majority of the included studies (25 out of 29) reported a statistically significant reduction in preoperative anxiety levels following the implementation of VR interventions. These results suggest a generally positive trend in favor of VR as an effective non-pharmacological approach for managing anxiety in surgical patients. However, four studies did not demonstrate statistically significant differences between the intervention and control groups. Figure 3 provides a schematic diagram that visually represents this variation in outcomes and highlights the distribution of results across the included studies.

## 4. Discussion

### 4.1. Summary of Findings

This study aimed to investigate the effect and effectiveness of VR technology in the management of pre-operative anxiety in adult patients who will undergo surgery. The results of the review indicate that VR can effectively provide patients with the necessary knowledge and information throughout the pre- and post-operative phases. Patients are supported in coping with surgical stress through user-friendly educational design. In addition, within a VR environment, patients can experience the surgical procedure and to immerse themselves in it; these familiarization processes bring physiological responses. VR distraction works by shifting the patient’s focus away from the stressor toward neutral or calming stimuli. The neurobiological systems involved in anxiety may be directly or indirectly affected by this distraction [51].

The prospective controlled observational study by Bidgoli et al. provides a direct comparison between an in-person operating room visit group, a VR operating room ‘live’ video visit group, and a standard care control group. Their analysis revealed no statistically significant difference in preoperative anxiety scores across these groups, measured both pre- and post-intervention [24].

In both the studies by Akar et al. (2024) and El-Mathari et al., the absence of statistically significant differences in anxiety reduction between the VR and control groups may be attributed to low baseline anxiety levels among participants. In the study by Akar et al., although surgical fear assessed by the SFQ scale showed significantly lower scores in the VR group at the third time point (operating theatre waiting room), no significant difference was found between groups in the VAS-A anxiety scores. Notably, both groups exhibited low initial anxiety levels (VAS-A ≤ 3), well below the commonly recognized threshold for clinically relevant anxiety (e.g., VAS-A ≥ 7 in cardiac surgery patients) [48,50].

Similarly, in the study by El-Mathari et al., participants were classified as anxious based on established cut-off values (STAI ≥ 40, APAIS ≥ 11), yet the average scores were below these thresholds, indicating limited baseline anxiety despite patients expressing concerns about the surgery. These findings highlight the potential influence of cultural and contextual factors in the perception and reporting of anxiety. They also underscore the importance of targeted use of VR interventions, particularly in patients with clinically significant preoperative anxiety, where the potential for measurable benefit is greater [50].

A meta-analysis by Koo et al. found that the anxiolytic effects of VR were more pronounced in pediatric patients than in adults [52]. The lack of significant anxiety reduction in adults may be due to the small number of studies and the differing psychological profiles between children and adults. These findings underscore the importance of considering patient age and psychological characteristics when evaluating VR effectiveness, emphasizing the need for comparable anxiety profiles across study groups to ensure valid outcome comparisons.

Drozdova et al. found no statistically significant difference in the quality of education between the VR educational group and the group that received information from a physician. In contrast, the study showed a significant reduction in preoperative anxiety in 92% of patients (*n* = 69) [23].

While the findings of this review generally support the effectiveness of VR in reducing preoperative anxiety, several sources of bias may influence these outcomes. Publication bias is a potential concern, as studies reporting statistically significant results are more likely to be published and indexed. Additionally, novelty bias may play a role, as the innovative and high-tech nature of VR could heighten participants’ interest and engagement, thereby amplifying perceived benefits. Placebo effects should also be considered, particularly in studies without blinding or comparator arms, where improvements in anxiety may result from positive expectations or increased attention rather than the VR content itself.

In order to verify the reduction of preoperative anxiety with the VR intervention, further RCT studies with homogeneous surgical populations need to be designed, considering the recommendations of previous studies in order to demonstrate the results in more concrete terms. Some recommendations that have emerged from the previous studies affect the effectiveness of VR, concerning the type of surgery that needs to be separated into minor and major, the type of intervention (exposure/distraction), the duration of exposure > 15 min, and the proper time for VR to be implemented preoperatively. Moreover, the findings emphasize the need for pre-surgical anxiety assessment so that VR interventions can be selectively implemented in patients presenting with high or very high levels of anxiety, where they are likely to be most beneficial.

### 4.2. Features of Interventions

Regarding the timing of exposure, the preoperative period is notably prolonged for patients undergoing elective surgery. This period typically begins at the time of surgical consent, often days before the procedure, and extends up to the moment of entry into the operating room. As a result, these patients are exposed to anxiety for a longer duration compared to other surgical patient categories. Among the included studies, the timing of VR intervention varied widely, ranging from several days prior to surgery to administration in the preoperative waiting area. The duration of VR in the studies appears to range from 3 to 35 min; the optimal length of intervention needs to be clarified. The frequency of intervention was also reported only once in the majority of studies.

It must also be considered that patients who have undergone surgery in the past have lower levels of pre-operative anxiety due to their previous exposure to the procedure and familiarity with it. Female patients and patients without previous surgical experience have higher levels of anxiety [5,53].

Important findings from meta-analyses by Chan et al. and Gao et al. help to emphasize that VR exposure education seems to be more effective than VR distraction intervention, and the reduction in preoperative anxiety symptom scores was higher when the enhanced VR intervention was patient-selected. Moreover, according to their subgroup analysis, it has been revealed that interventions chosen based on patient preferences are the most effective strategy. When patients can choose the intervention type, the intervention is more effective in reducing preoperative anxiety [54,55]. VR can also be used as a preventive strategy to improve psychological well-being and to stabilize the vital signs of patients undergoing surgery [54].

Patients’ preoperative anxiety symptoms may be influenced by different cultural backgrounds [56]. According to the World Health Organization, Asia has fewer doctors per 10,000 patients. The high demand for medical personnel leads to a culture of short medical examinations [57]. A study has shown that patients in Asia had reduced levels of preoperative anxiety after VR intervention [54]. The above data are confirmed by the meta-analysis of Chan et al., who considered that another possible reason for the effectiveness of VR is the reduced duration of the medical examination [54] and believe that educational exposure to VR can fill the gap by providing preoperative information.

The studies conducted by Xu et al. and Asiri et al. highlight the positive impact of VR on patient satisfaction [47,58]. Specifically, Asiri et al. carried out a RCT involving a substantial number of participants (n = 98), demonstrating the effectiveness of VR not only in reducing preoperative anxiety, which they emphasized should be assessed prior to the implementation of the technology, but also in enhancing the overall patient experience, as evidenced by a reduced length of stay (LOS) and increased patient satisfaction [47]. Similarly, Xu et al., through a meta-analysis, confirmed that the use of VR can significantly improve patient satisfaction following surgical procedures, further supporting the potential benefits of VR beyond anxiety reduction [58].

We await with great interest the results of the RCT study by Gabalawy et al. (a three-phase development and feasibility trial, investigating “Immersive VR Intervention for Preoperative Anxiety and Distress Among Adults Undergoing Oncological Surgery (Breast cancer), Canada 2024)” [59]. This randomized feasibility study evaluates the novel VR preoperative intervention compared to a VR control condition (i.e., nature trek) and a routine treatment group in patients undergoing breast cancer surgery. Data analysis is ongoing in Phase 3.

Of particular interest were the results of the study by Schmid et al., in which VR was applied to manage preoperative anxiety in patients undergoing surgery for gynecological cancers. Conducting a study on a population that exhibits two pivotal characteristics conducive to elevated anxiety from the outset, such as the female gender itself and cancer, renders the study arduous and the outcomes thereof unpredictable. Oncology patients are perhaps the most challenging population to manage anxiety in the preoperative setting because of the survival issues they are facing. However, a significant decrease in anxiety was observed for the VR educational intervention group (three-dimensional virtual tour). VR had a lasting impact on reducing anxiety, even after a prolonged period had passed before the surgery was performed, 35 (15–53) days before surgery. Age, procedure type, and period between anxiety assessments had no impact. It seems that the effectiveness of VR in the reduction of anxiety is due to its provision of a sense of control and predictability to patients, with the resultant alleviation of the uncertainty associated with surgery. VR can promote a more positive mindset in patients going into surgery by reducing anticipatory anxiety and preventing negative thought patterns [49].

### 4.3. Postoperative Pain Management

According to postoperative pain management by VR intervention in the studies reviewed, only the RCT by Abbasnia et al. measured the effectiveness of VR in postoperative pain management, but they applied VR not only before surgery, a method that other studies followed, but VR was also applied 4 h after surgery, a fact that positively affects the perception of pain and reduce it [33]. In the meta-analysis of Ding et al., the results showed a greater reduction in postoperative pain in patients who received VR intraoperatively and postoperatively, compared with usual care. No significant reduction in postoperative pain relief was found when patients received VR during the preoperative period. The effect of preoperative VR is not continuous and, therefore, may not be effective in influencing pain perception postoperatively. The importance of using VR at the right time is underlined by this finding [60]. In addition, the VR intervention decreases systolic blood pressure and heart rate, but not diastolic blood pressure. White coat syndrome, which affects pre- and post-operative vital sign measurements, may explain this result [54].

### 4.4. Effectiveness of VR on Anxiety

VR is a practical and cost-effective tool that can provide long-term efficiency benefits in clinical settings. Healthcare professionals can use VR as a proactive strategy to implement preoperative education, promote psychological well-being, and stabilize the vital signs of patients undergoing surgery, enabling them to be satisfied and allowing them to devote time to other tasks. At the same time, patients can receive preoperative education through VR that is tailored to their needs, providing them with all the information they need in detail, with the ability to re-view the content whenever they wish.

VR may also improve the patient’s postoperative course, such as recovery time, expected healing of the surgical wound, desired therapeutic outcome, improvement of vital signs (e.g., blood pressure, heart rate), better management of postoperative pain, and reduction of infections. It is a non-invasive and non-pharmacological intervention with minimal and rare side effects [9,61,62,63].

To integrate the use of VR technology into the preoperative care process, it is necessary to establish specifications concerning the characteristics of the method. It is also necessary to determine the optimal time of use of the device, which will make the method effective in reducing preoperative anxiety, always following the recommendations of the guidelines for these devices in terms of safety of use for patients. Finally, further research is needed to determine which types of VR content are most effective in reducing anxiety across patient groups.

Even though the present systematic review focused on VR, it is important to note that other non-pharmacological interventions such as music therapy, guided imagery, and cognitive behavioral therapy have also shown efficacy in reducing preoperative anxiety. Despite VR offering particular advantages—including immersive engagement and interactive customization—direct comparisons of VR with these established interventions are currently restricted. Future research should aim to compare VR directly with other psychological strategies to determine relative effectiveness and cost–benefit profiles.

### 4.5. Strengths and Limitations

This systematic review offers valuable insights into the role of VR as a non-pharmacological intervention for managing preoperative anxiety. A key strength of the study is the inclusion of 19 RCTs, which provide robust evidence supporting the effectiveness of VR in clinical settings. The use of validated anxiety measurement scales (e.g., STAI, VAS, HADS-A) enables a standardized assessment of outcomes, enhancing the reliability of the findings. VR’s potential to offer a safe, controlled, and immersive environment for anxiety reduction may reduce the reliance on pharmacological agents, lowering healthcare costs and minimizing side effects. Furthermore, investigating the clinical application of VR represents an innovative approach with promising implications for perioperative care.

Despite its strengths, the review is subject to several limitations. The search was restricted to English-language publications, potentially excluding relevant studies in other languages. Significant heterogeneity across the included studies, regarding intervention protocols (timing, duration, and content), surgical procedures, anesthesia types, and patient demographics (age, gender, health status, and cultural background), compromised data comparability and limited generalizability. The inconsistent reporting of anesthetic techniques and variability in anxiety assessment tools added further complexity. The wide range in sample sizes (1 to 150 participants) may also impact the robustness of conclusions. Due to these methodological discrepancies, particularly in outcome reporting (i.e., means vs medians, standard deviations vs interquartile ranges), a meta-analysis was deemed inappropriate; thus, a narrative synthesis was employed. Moreover, the absence of control groups in some studies restricts the ability to isolate the effects of VR interventions.

Future research should aim for greater methodological standardization, consistent reporting practices, and well-defined clinical settings to strengthen the evidence base and support the broader clinical adoption of VR in perioperative care.

## 5. Conclusions

Based on the anxiety measurement scales applied in each study, the majority of cases demonstrated a statistically significant reduction in preoperative anxiety following the use of VR interventions. Considering the results of the present systematic review, VR technology appears to be a promising non-pharmacological tool in the management of preoperative anxiety by improving patients’ well-being before surgical procedures.

Beyond the psychological benefits, VR could also offer additional advantages. Practically, it could reduce the need for higher doses of anesthetics and analgesics. Reduced anxiety before surgery may contribute indirectly to better perioperative outcomes, including fewer complications and decreased healthcare utilization, though this was not directly assessed in this review. From an economic perspective, these factors collectively may contribute to more effective perioperative care and could lead to a reduction in overall healthcare costs in the perioperative setting, potentially reducing the reliance on pharmacological interventions.

Further research is warranted to rigorously evaluate the efficacy of VR interventions, particularly through well-designed, large-scale RCTs involving homogeneous patient populations. Such studies are essential to determine the optimal use of VR in perioperative settings and to inform the development of standardized clinical protocols. Establishing a strong evidence base will be essential for the wider integration of VR as a reliable and effective approach to managing preoperative anxiety in clinical practice.

## Figures and Tables

**Table 1:** Summary of quality appraisal of studies using the MMAT (Mixed Methods Appraisal Tool).

Assessment Criteria																	
			All Studies		Quant. Randomized Controlled Trials					Quant. Non -Randomized Controlled Trials							
	Author	Study Type	S1. Are There Clear Research Questions?	S2. Do the Collected Data Allow to Address the Research Questions?	2.1. Is Randomization Appropriately Performed?	2.2. Are the Groups Comparable at Baseline?	2.3. Are There Complete Outcome Data?	2.4. Are Outcome Assessors Blinded to the Intervention Provided?	2.5 Did the Participants Adhere to the Assigned Intervention?	3.1. Are the Participants Representative of the Target Population?	3.2. Are Measurements Appropriate Regarding Both the Outcome and Intervention (or Exposure)?	3.3. Are There Complete Outcome Data?	3.4. Are the Confounders Accounted for in the Design and Analysis?	3.5. During the Study Period, is the Intervention Administered (or Exposure Occurred) as Intended?	Total Metrics	Score %	Risk of Bias
1	Drozdova et al. [23]	RCT	Y	Y	Y	Y	Y	N	Y						4/5	80%	Moderate
2	Bidgoli et al. [24]	Quant non-RCT	Y	Y						Y	Y	Y	CT	Y	4/5	80%	Moderate
3	Amiri et al. [25]	Quant non-RCT	Y	Y						Y	Y	Y	N	Y	4/5	80%	Moderate
4	Pandrangi et al. [26]	RCT	Y	Y	Y	Y	Y	CT	Y						4/5	80%	Moderate
5	Liu Y et al. [27]	RCT	Y	Y	Y	Y	Y	Y	Y						5/5	100%	Low
6	Rougereau G et al. [28]	RCT	Y	Y	Y	Y	Y	N	Y						4/5	80%	Moderate
7	Grab M et al. [29]	RCT	Y	Y	CT	Y	Y	Y	Y						4/5	80%	Moderate
8	Kwon H et al. [30]	RCT	Y	Y	Y	Y	Y	Y	Y						5/5	100%	Low
9	Flores A. et al. [31]	Case Report	Y	Y						Y	Y	CT	CT	Y	3/5	60%	Moderate
10	Martinez-Bernal D. [32]	Quant non-RCT	Y	Y						Y	Y	Y	CT	Y	4/5	80%	Moderate
11	Abbasnia F. et al. [33]	RCT	Y	Y	Y	Y	Y	Y	Y						4/5	80%	Moderate
12	Hermans ANL et al. [34]	Quant non-RCT	Y	Y						Y	Y	Y	CT	Y	4/5	80%	Moderate
13	Ugras GA. et al. [35]	RCT	Y	Y	Y	Y	Y	CT	Y						4/5	80%	Moderate
14	Chiu et al. [15]	RCT	Y	Y	Y	Y	Y	Y	Y						5/5	100%	Low
15	Aardoom JJ. et al. [36]	Quanti. Descr.	Y	Y						Y	Y	N	N	Y	3/5	60%	Moderate
16	Touil N. et al. [37]	Quant non-RCT	Y	Y						Y	Y	Y	CT	Y	4/5	80%	Moderate
17	Baytar AD. et al. [38]	Quant non-RCT	Y	Y						Y	Y	Y	CT	Y	4/5	80%	Moderate
18	Turrado V. et al. [39]	RCT	Y	Y	Y	Y	Y	N	Y						4/5	80%	Moderate
19	Turan AZ. et al. [40]	RCT	Y	Y	Y	Y	Y	N	Y						4/5	80%	Moderate
20	Chan JJI. et al. [41]	Quant non-RCT	Y	Y						Y	Y	Y	CT	Y	4/5	80%	Moderate
21	Hendricks et al. [42]	RCT	Y	Y	Y	Y	Y	CT	Y						5/5	80%	Moderate
22	Noben L. et al. [43]	RCT	Y	Y	Y	Y	Y	N	Y						4/5	80%	Moderate
23	Ganry L. et al. [44]	Quant non-RCT	Y	Y						Y	Y	Y	CT	Y	4/5	80%	Moderate
24	Bekelis K. et al. [45]	RCT	Y	Y	Y	Y	Y	N	Y						4/5	80%	Moderate
25	Wang Y et al. [46]	RCT	Y	Y	Y	Y	Y	N	Y						4/5	80%	Moderate
26	Asiri et al. [47]	RCT	Y	Y	Y	Y	Y	N	Y						4/5	80%	Moderate
27	Akar et al. [48]	RCT	Y	Y	Y	Y	Y	Y	Y						5/5	100%	Low
28	Schmid et al. [49]	RCT	Y	Y	Y	Y	Y	N	Y						4/5	80%	Moderate
29	El Mathari et al. [50]	RCT	Y	Y	Y	Y	Y	N	Y						4/5	80%	Moderate

Note. Y: yes (if it met quality criterion); N: no (if it did not meet quality criterion); CT: cannot tell (if it did not mention relevant information); Quant non-RCT: quantitative non-randomized control study.

## Public Involvement Statement

No public involvement in any aspect of this research.

## Guidelines and Standards Statement

This manuscript was drafted against PRISMA MODEL for Systematic Reviews.

## Abbreviations

The following abbreviations are used in this manuscript:

PRISMA	Preferred Reporting Items for Systematic Reviews and Meta-Analyses
RCT	Randomized Controlled Trial
VR	Virtual Reality

## References

[1] Weiser T.G., Haynes A.B., Molina G., Lipsitz S.R., Esquivel M.M., Uribe-Leitz T., Fu R., Azad T., Chao T.E., Berry W.R. (2016). Size and distribution of the global volume of surgery in 2012. Bull. World Health Organ..

[2] Stamenkovic D.M., Rancic N.K., Latas M.B., Neskovic V., Rondovic G.M., Wu J.D., Cattano D. (2018). Preoperative anxiety and implications on postoperative recovery: What can we do to change our history. Minerva Anestesiol..

[3] Ki M., Kim D.-C., You S.W., Oh J., Jang J., Yoo H.H. (2023). Appropriateness of the anxiety subscale of the Hospital Anxiety and Depression Scale for Koreans to measure preoperative anxiety and the effect of preoperative anxiety on postoperative quality of recovery. Anesth. Pain Med..

[4] Ji W., Sang C., Zhang X., Zhu K., Bo L. (2022). Personality, Preoperative Anxiety, and Postoperative Outcomes: A Review. Int. J. Environ. Res. Public. Health.

[5] Dibabu A.M., Ketema T.G., Beyene M.M., Belachew D.Z., Abocherugn H.G., Mohammed A.S. (2023). Preoperative anxiety and associated factors among women admitted for elective obstetric and gynecologic surgery in public hospitals, Southern Ethiopia: A cross-sectional study. BMC Psychiatry.

[6] Ben-Arye E., Segev Y., Galil G., Marom I., Gressel O., Stein N., Hirsh I., Samuels N., Schmidt M., Schiff E. (2023). Acupuncture during gynecological oncology surgery: A randomized controlled trial assessing the impact of integrative therapies on perioperative pain and anxiety. Cancer.

[7] El Mathari S., Hoekman A., Kharbanda R.K., Sadeghi A.H., Wijngaarden R.d.L.v., Götte M., Klautz R.J., Kluin J. (2024). Virtual Reality for Pain and Anxiety Management in Cardiac Surgery and Interventional Cardiology. JACC Adv..

[8] Tully P.J., Baker R.A., Knight J.L. (2008). Anxiety and depression as risk factors for mortality after coronary artery bypass surgery. J. Psychosom. Res..

[9] Kiecolt-Glaser J.K., Page G.G., Marucha P.T., MacCallum R.C., Glaser R. (1998). Psychological Influences on Surgical Recovery: Perspectives from psychoneuroimmunology. Am. Psychol..

[10] Moline L.R. (2000). Patient psychologic preparation for invasive procedures: An integrative review. J. Vasc. Nurs..

[11] Perks A.M., Chakravarti S., Manninen P.M. (2009). Preoperative Anxiety in Neurosurgical Patients. J. Neurosurg. Anesthesiol..

[12] Levandovski R., Ferreira M.B.C., Hidalgo M.P.L., Konrath C.A., da Silva D.L., Caumo W. (2008). Impact of preoperative anxiolytic on surgical site infection in patients undergoing abdominal hysterectomy. Am. J. Infect. Control..

[13] Attias S., Boker L.K., Arnon Z., Ben-Arye E., Bar’AM A., Sroka G., Matter I., Somri M., Schiff E. (2016). Effectiveness of integrating individualized and generic complementary medicine treatments with standard care versus standard care alone for reducing preoperative anxiety. J. Clin. Anesth..

[14] Duncan A.E. (2012). Hyperglycemia and perioperative glucose management. Curr. Pharm. Des..

[15] Chiu P.L., Li H., Yap K.Y.-L., Lam K.-M.C., Yip P.-L.R., Wong C.L. (2023). Virtual Reality–Based Intervention to Reduce Preoperative Anxiety in Adults Undergoing Elective Surgery: A Randomized Clinical Trial. JAMA Netw. Open.

[16] Ahmadpour N., Randall H., Choksi H., Gao A., Vaughan C., Poronnik P. (2019). Virtual Reality interventions for acute and chronic pain management. Int. J. Biochem. Cell Biol..

[17] Li L., Yu F., Shi D., Shi J., Tian Z., Yang J., Wang X., Jiang Q. (2017). Application of virtual reality technology in clinical medicine. Am. J. Transl. Res..

[18] Anthes C., Garcia-Hernandez R.J., Wiedemann M., Kranzlmuller D. (2016). State of the art of virtual reality technology. Proceedings of the 2016 IEEE Aerospace Conference.

[19] Monash University, Health, Safety and Wellbeing (2022). Safe Use of Immersive Technologies: Virtual Reality (VR), Augmented Reality (AR), Mixed Reality (MR) Guidelines, v1.0 [Internet]. http://www.monash.edu.au/ohs/.

[20] Palmer J.A. (2007). Decreasing Anxiety Through Patient Education. Plast. Surg. Nurs..

[21] Atkinson L.Z., Cipriani A. (2018). How to carry out a literature search for a systematic review: A practical guide. BJPsych Adv..

[22] Hong Q.N., Fàbregues S., Bartlett G., Boardman F., Cargo M., Dagenais P., Gagnon M.-P., Griffiths F., Nicolau B., O’Cathain A. (2018). The Mixed Methods Appraisal Tool (MMAT) version 2018 for information professionals and researchers. Educ. Inf..

[23] Drozdova A., Polokova K., Jiravsky O., Godula B.J., Chovancik J., Ranic I., Jiravsky F., Hecko J., Sknouril L. (2024). Comparing Conventional Physician-Led Education with VR Education for Pacemaker Implantation: A Randomized Study. Healthcare.

[24] Bidgoli Z.A., Sadat Z., Zarei M., Ajorpaz N.M., Hosseinian M. (2023). Does a 30-minute introductory visit to the operating room reduce patients’ anxiety before elective surgery? A prospective controlled observational study. Patient Saf. Surg..

[25] Amiri A., Jalali R., Salari N. (2023). The effect of using virtual reality technology on anxiety and vital signs before surgery in patients undergoing open heart surgery. Perioper. Med..

[26] Pandrangi V.C., Low G., Slijepcevic A., Shah S., Shindo M., Schindler J., Colaianni A., Clayburgh D., Andersen P., Flint P. (2024). Use of Perioperative Virtual Reality Experiences on Anxiety and Pain: A Randomized Comparative Trial. Laryngoscope.

[27] Liu Y., Wang R., Zhang Y., Feng L., Huang W. (2023). Virtual reality psychological intervention helps reduce preoperative anxiety in patients undergoing carotid artery stenting: A single-blind randomized controlled trial. Front. Psychol..

[28] Rougereau G., Sandiford M.H., Lévêque R., Ménigaux C., Bauer T., Hardy A. (2023). Management of Anxiety for Ambulatory Hallux Valgus Surgery with a Virtual Reality Hypnosis Mask: Randomized Controlled Trial. Foot Ankle Int..

[29] Grab M., Hundertmark F., Thierfelder N., Fairchild M., Mela P., Hagl C., Grefen L. (2023). New perspectives in patient education for cardiac surgery using 3D-printing and virtual reality. Front. Cardiovasc. Med..

[30] Kwon H., Lee J., Park Y.S., Oh S.-H., Kim J. (2023). Effects of preoperative education using virtual reality on preoperative anxiety and information desire: A randomized clinical trial. J. Clin. Monit. Comput..

[31] Flores A., Hoffman H.G., Navarro-Haro M.V., Garcia-Palacios A., Atzori B., Le May S., Alhalabi W., Sampaio M., Fontenot M.R., Mason K.P. (2023). Using Immersive Virtual Reality Distraction to Reduce Fear and Anxiety before Surgery. Healthcare.

[32] Martinez-Bernal D., Cross W.F., Hasselberg M., Tapparello C., Stenz C.F.H., Kolokythas A. (2023). A brief virtual reality intervention for pre-operative anxiety in adults. Oral Surg. Oral Med. Oral Pathol. Oral Radiol..

[33] Abbasnia F., Aghebati N., Miri H.H., Etezadpour M. (2023). Effects of Patient Education and Distraction Approaches Using Virtual Reality on Pre-operative Anxiety and Post-operative Pain in Patients Undergoing Laparoscopic Cholecystectomy. Pain Manag. Nurs..

[34] Hermans A.N.L., Betz K., Verhaert D.V.M., Uijl D.W.D., Clerx K., Debie L., Lahaije M., Vernooy K., Linz D., Weijs B. (2023). 360° Virtual reality to improve patient education and reduce anxiety towards atrial fibrillation ablation. EP Eur..

[35] Ugras G.A., Kanat C., Yaman Z., Yilmaz M., Turkmenoglu M.O. (2023). The Effects of Virtual Reality on Preoperative Anxiety in Patients Undergoing Colorectal and Abdominal Wall Surgery: A Randomized Controlled Trial. J. Perianesth. Nurs..

[36] Aardoom J.J., Hilt A.D., Woudenberg T., Chavannes N.H., E Atsma D. (2022). A Preoperative Virtual Reality App for Patients Scheduled for Cardiac Catheterization: Pre–Post Questionnaire Study Examining Feasibility, Usability, and Acceptability. JMIR Cardio.

[37] Touil N., Pavlopoulou A., Momeni M., Van Pee B., Barbier O., Sermeus L., Roelants F. (2021). Evaluation of virtual reality combining music and a hypnosis session to reduce anxiety before hand surgery under axillary plexus block: A prospective study. Int. J. Clin. Pract..

[38] Baytar Ç., Bollucuoğlu K. (2023). Effect of virtual reality on preoperative anxiety in patients undergoing septorhinoplasty. Braz. J. Anesthesiol..

[39] Turrado V., Guzmán Y., Jiménez-Lillo J., Villegas E., De Lacy F.B., Blanch J., Balibrea J.M., Lacy A. (2021). Exposure to virtual reality as a tool to reduce peri-operative anxiety in patients undergoing colorectal cancer surgery: A single-center prospective randomized clinical trial. Surg. Endosc..

[40] Turan A.Z., Yilmaz M., Saracoglu T. (2021). The effect of virtual reality glasses on anxiety during surgery under spinal anesthesia: A randomized controlled study. Anaesth. Pain Intensive Care.

[41] Chan J.J.I., Yeam C.T., Kee H.M., Tan C.W., Sultana R., Sia A.T.H., Sng B.L. (2020). The use of pre-operative virtual reality to reduce anxiety in women undergoing gynecological surgeries: A prospective cohort study. BMC Anesthesiol..

[42] Hendricks T.M., Gutierrez C.N., Stulak J.M., Dearani J.A., Miller J.D. (2020). The Use of Virtual Reality to Reduce Preoperative Anxiety in First-Time Sternotomy Patients: A Randomized Controlled Pilot Trial. Mayo Clin. Proc..

[43] Noben L., Goossens S.M.T.A., Truijens S.E.M., Van Berckel M.M.G., Perquin C.W., Slooter G.D., Van Rooijen S.J. (2019). A Virtual Reality Video to Improve Information Provision and Reduce Anxiety Before Cesarean Delivery: Randomized Controlled Trial. JMIR Ment. Health.

[44] Ganry L., Hersant B., Sidahmed-Mezi M., Dhonneur G., Meningaud J.P. (2018). Using virtual reality to control preoperative anxiety in ambulatory surgery patients: A pilot study in maxillofacial and plastic surgery. J. Stomatol. Oral Maxillofac. Surg..

[45] Bekelis K., Calnan D., Simmons N., MacKenzie T.A., Kakoulides G. (2017). Effect of an Immersive Preoperative Virtual Reality Experience on Patient Reported Outcomes: A Randomized Controlled Trial. Ann. Surg..

[46] Wang Y., Sun J., Yu K., Liu X., Liu L., Miao H., Li T. (2024). Virtual reality exposure reduce acute postoperative pain in female patients undergoing laparoscopic gynecology surgery: A Randomized Control Trial (RCT) study. J. Clin. Anesth..

[47] Asiri S., Guilhermino M., Duff J., Currie J., Guilhermino M. (2024). The Effectiveness of Virtual Reality Technology for Perioperative Anxiety Among Adults Undergoing Elective Surgery. Ph.D. Thesis.

[48] Akar T.E., Ünver S. (2024). Effectiveness of Virtual Reality Glasses on Surgical Fear and Anxiety in Patients Before Open-heart Surgery: A Double-blind Randomized Controlled Trial. J. Perianesth. Nurs..

[49] Schmid B.C., Marsland D., Jacobs E., Rezniczek G.A. (2024). A Preparatory Virtual Reality Experience Reduces Anxiety before Surgery in Gynecologic Oncology Patients: A Randomized Controlled Trial. Cancers.

[50] El Mathari S., Kuitert L., Boulidam N., Shehadeh S., Klautz R.J.M., de Lind van Wijngaarden R., Kluin J. (2024). Evaluating Virtual Reality Patient Education in Cardiac Surgery: Impact on Preoperative Anxiety and Postoperative Patient Satisfaction. J. Clin. Med..

[51] Wu J., Yan J., Zhang L., Chen J., Cheng Y., Wang Y., Zhu M., Cheng L., Zhang L. (2022). The effectiveness of distraction as preoperative anxiety management technique in pediatric patients: A systematic review and meta-analysis of randomized controlled trials. Int. J. Nurs. Stud..

[52] Koo C.-H., Park J.-W., Ryu J.-H., Han S.-H. (2020). The Effect of Virtual Reality on Preoperative Anxiety: A Meta-Analysis of Randomized Controlled Trials. J. Clin. Med..

[53] Caumo W., Schmidt A.P., Schneider C.N., Bergmann J., Iwamoto C.W., Bandeira D., Ferreira M.B.C. (2001). Risk factors for preoperative anxiety in adults. Acta. Anaesthesiol Scand..

[54] Chan S.L., Sit J.W.H., Ang W.W., Lau Y. (2024). Virtual reality-enhanced interventions on preoperative anxiety symptoms in adults undergoing elective surgery: A meta-analysis and meta-regression. Int. J. Nurs. Stud..

[55] Gao Y., Wang N., Liu N. (2023). Effectiveness of virtual reality in reducing preoperative anxiety in adults: A systematic review and meta-analysis. J. Adv. Nurs..

[56] Lazarus R.S., Folkman S. (1987). Transactional theory and research on emotions and coping. Eur. J. Personal..

[57] Deveugele M., Derese A., van den Brink-Muinen A., Bensing J., De Maeseneer J. (2022). Consultation length in general practice: Cross sectional study in six European countries. BMJ.

[58] Xu H., Hou J., Zhou J., Wang S. (2024). Effects of Virtual Reality on Preoperative Anxiety in Adult Patients: An Updated Meta-analysis. J. Perianesth Nurs..

[59] El-Gabalawy R., Sommer J.L., Hebbard P., Reynolds K., Logan G.S., Smith M.S.D., Mutter T.C., Mutch W.A., Mota N., Proulx C. (2024). An Immersive Virtual Reality Intervention for Preoperative Anxiety and Distress Among Adults Undergoing Oncological Surgery: Protocol for a 3-Phase Development and Feasibility Trial. JMIR Res. Protoc..

[60] Ding L., Hua H., Zhu H., Zhu S., Lu J., Zhao K., Xu Q. (2020). Effects of virtual reality on relieving postoperative pain in surgical patients: A systematic review and meta-analysis. Int. J. Surg..

[61] Bayrak A., Sagiroglu G., Copuroglu E. (2019). Effects of Preoperative Anxiety on Intraoperative Hemodynamics and Postoperative Pain. J. Coll. Phys. Surg. Pak..

[62] Chen Y.-Y.K., Soens M.A., Kovacheva V.P. (2022). Less stress, better success: A scoping review on the effects of anxiety on anesthetic and analgesic consumption. J. Anesth..

[63] Agüero-Millan B., Abajas-Bustillo R., Ortego-Maté C. (2023). Efficacy of nonpharmacologic interventions in preoperative anxiety: A systematic review of systematic reviews. J. Clin. Nurs..

